# DeepPurpose: a deep learning library for drug–target interaction prediction

**DOI:** 10.1093/bioinformatics/btaa1005

**Published:** 2020-12-12

**Authors:** Kexin Huang, Tianfan Fu, Lucas M Glass, Marinka Zitnik, Cao Xiao, Jimeng Sun

**Affiliations:** btaa1005-aff1 Harvard University, Boston, MA 02115, USA; btaa1005-aff2 Georgia Institute of Technology, Atlanta, GA 30332, USA; btaa1005-aff3 IQVIA, Cambridge, MA 02139, USA; btaa1005-aff4 University of Illinois at Urbana-Champaign, Urbana, IL 61801, USA

## Abstract

**Summary:**

Accurate prediction of drug–target interactions (DTI) is crucial for drug discovery. Recently, deep learning (DL) models for show promising performance for DTI prediction. However, these models can be difficult to use for both computer scientists entering the biomedical field and bioinformaticians with limited DL experience. We present DeepPurpose, a comprehensive and easy-to-use DL library for DTI prediction. DeepPurpose supports training of customized DTI prediction models by implementing 15 compound and protein encoders and over 50 neural architectures, along with providing many other useful features. We demonstrate state-of-the-art performance of DeepPurpose on several benchmark datasets.

**Availability and implementation:**

https://github.com/kexinhuang12345/DeepPurpose.

**Supplementary information:**

Supplementary data are available at *Bioinformatics* online.

## 1 Introduction

Drug–target interactions (DTI) characterize the binding of compounds to protein targets (Santos *et al.*, 2017). Accurate identification of molecular drug targets is fundamental for drug discovery and development (Rutkowska *et al.*, 2016; Zitnik *et al.*, 2019) and is especially important for finding effective and safe treatments for new pathogens, including SARS-CoV-2 (Velavan and Meyer, 2020).

Deep learning (DL) has advanced traditional computational modeling of compounds by offering an increased expressive power in identifying, processing and extrapolating complex patterns in molecular data (Lee *et al.*, 2019; Öztürk *et al.*, 2018). There are many DL models designed for DTI prediction (Lee *et al.*, 2019; Nguyen *et al.*, 2020; Öztürk *et al.*, 2018). However, to generate predictions, deploy DL models in practice, test and evaluate model performance, one needs considerable programming skills and extensive biochemical knowledge. Prevailing tools are designed for experienced interdisciplinary researchers. They are challenging to use by both computer scientists entering the biomedical field and domain bioinformaticians with limited experience in training and deploying DL models. Furthermore, each open-sourced tool has a different programming interface and is coded differently, which prevents easy integration of outputs from various methods for model ensembles (Yang *et al.*, 2019).

Here, we introduce DeepPurpose, a DL library for encoding and downstream prediction of proteins and compounds. DeepPurpose allows rapid prototyping via a programming framework that implements over 50 DL models, seven protein encoders and eight compound encoders. Empirically, we find that models implemented in DeepPurpose achieve state-of-the-art prediction performance on DTI benchmark datasets.

## 2 DeepPurpose library

DL models for DTI prediction can be formulated as an encoder-decoder architectures (Cho *et al.*, 2014). DeepPurpose library implements a unifying encoder-decoder framework, which makes the library uniquely flexible. By merely specifying an encoder’s name, the user can automatically connect a encoder of interest with the relevant decoder. DeepPurpose then trains the corresponding encoder-decoder model in an end-to-end manner. Finally, the user accesses the trained model either programmatically or via a visual interface and uses the model for DTI prediction.

### 2.1 Module for encoding proteins and compounds

DeepPurpose takes the compound’s simplified molecular-input line-entry system (SMILES) string and protein amino acid sequence pair as input. Then, they are fed into molecular encoders which specifies a deep transformation function that maps compounds and proteins to a vector representation. In particular, for compounds, DeepPurpose provides eight encoders using different modalities of compounds: Multi-Layer Perceptrons (MLP) on Morgan, PubChem, Daylight and RDKit 2D Fingerprint; Convolutional Neural Network (CNN) on SMILES strings; Recurrent Neural Network (RNN) on top of CNN; transformer encoders on substructure fingerprints; message passing graph neural network on molecular graph. For proteins, DeepPurpose provides seven encoders for the input amino acid sequence: MLP on Amino Acid Composition (AAC), Pseudo AAC, Conjoint Triad, Quasi-Sequence descriptors; CNN on amino acid sequences; RNN on top of CNN; transformer encoder on substructure fingerprints. Note that alternative input features may not work for a specific encoder architecture. The detailed encoder specifications and references are described in Supplementary Material.

### 2.2 Module for DTI prediction

DeepPurpose feeds the learned protein and compound embeddings into an MLP decoder to generate predictions. Output scores include both continuous binding scores, such as the median inhibitory concentration (${\text{IC}}_{50}$), as well as binary outputs indicating whether a protein binds to a compound. The library detects whether the task is regression or classification and switches to the correct loss function and evaluation metrics. In the case of regression, we use the Mean Square Error (MSE) as the loss function and MSE, Concordance Index and Pearson Correlation as performance metrics. In the classification case, we use Binary Cross Entropy as the loss function and Area Under the Receiver Operating Characteristics (AUROC), Area Under Precision-Recall (AUPRC) and F-1 score as performance metrics. At inference, given new proteins and new compounds, DeepPurpose returns prediction scores representing predicted probabilities of binding between compounds and proteins.

### 2.3 Modules for other downstream prediction tasks

DeepPurpose includes repurposing and virtual_screening functions. Using only a few lines of codes that specify a list of compounds library to be screened upon and an optional set of training dataset, DeepPurpose trains five DL models, aggregates prediction results and generates a descriptive ranked list in which compound candidates with the highest predicted binding scores are placed at the top. If the user does not specify a training dataset, DeepPurpose uses a pre-trained deep model for prediction. This list can then be examined to identify promising compound candidates for further experiments. Second, DeepPurpose also supports user-friendly programming frameworks for other modeling tasks, including drug and protein property prediction, drug–drug interaction prediction and protein–protein interaction prediction (see Supplementary Material). Third, DeepPurpose provides an interface to many types of data, including public large binding affinity dataset (Liu *et al.*, 2007), bioassay data (Kim *et al.*, 2019) and a drug repurposing library (Corsello *et al.*, 2017).

### 2.4 Programming framework and implementation details

The functionality of DeepPurpose is modularized into six key steps where a single line of code can invoke each step: (i) Load the dataset from a local file or load a DeepPurpose benchmark dataset. (ii) Specify the names of compound and protein encoders. (iii) Split the dataset into training, validation and testing sets using data_process function, which implements a variety of data-split strategies. (iv) Create a configuration file and specify model parameters. If needed, DeepPurpose can automatically search for optimal values of hyper-parameters. (v) Initialize a model using the configuration file. Alternatively, the user can load a pre-trained model or a previously saved model. (vi) Finally, train the model using train function and monitor the progress of training and performance metrics. DeepPurpose is OS-agnostic and uses the Jupyter Notebook interface. It can be run in the cloud or locally. All datasets, models, documentation, installation instructions and tutorials are provided at https://github.com/kexinhuang12345/DeepPurpose.

## 3 Using DeepPurpose for DTI prediction

**Fig. 1.**
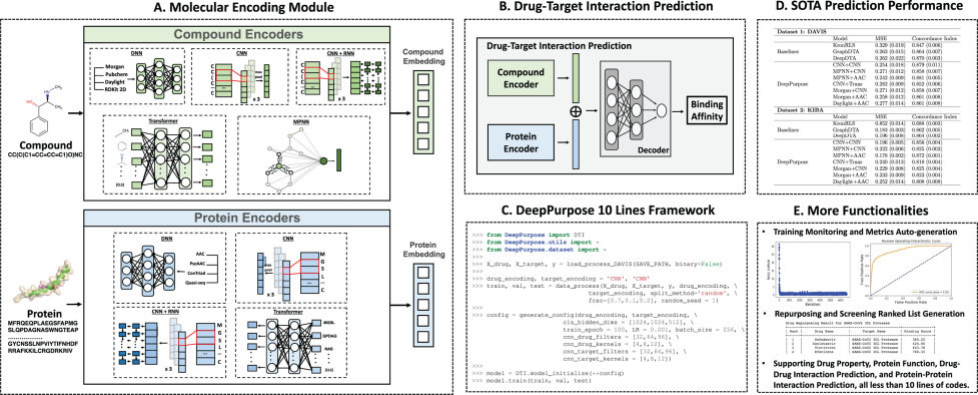
Overview of DeepPurpose library. (**A**) DeepPurpose takes as input the SMILES of a compound and a protein’s amino acid sequence and then generates embeddings for them. (**B**) The learned embeddings are then concatenated and fed into a decoder to predict DTI binding affinity. (**C**) DeepPurpose provides a simple but flexible programming framework that implements over 50 state-of-the-art DL models for DTI prediction. (**D**) DeepPurpose models achieve comparable performance with three other DTI prediction algorithms on two benchmark datasets. (**E**) Finally, DeepPurpose has many functionalities, including monitoring the training process, debugging and generation ranked lists for repurposing and screening. Further, DeepPurpose supports other downstream prediction tasks (e.g. drug–drug interaction prediction, compound property prediction)

To demonstrate the use of DeepPurpose, we compare DeepPurpose with KronRLS (Pahikkala *et al.*, 2015), a popular DTI method, and GraphDTA (Nguyen *et al.*, 2020) and DeepDTA (Öztürk *et al.*, 2018), state-of-the-art DL methods. We find that many DeepPurpose models achieve comparable prediction performance on two benchmark datasets, DAVIS (Davis *et al.*, 2011) and KIBA (He *et al.*, 2017) (Fig. 1D). A complete script to generate the results is provided in Supplementary Material.

## 4 DeepPurpose with interactive web interface

In addition to rapid model prototyping, DeepPurpose also provides utility functions to load a pre-trained model and make predictions for a new drug and target inputs. This functionality allows domain scientists to examine predictions quickly, modify the inputs based on predictions, and iterate on the process until finding a drug or target with desired properties. We leverage Gradio (Abid *et al.*, 2019) to create a web interface programmatically. We use a user-trained DeepPurpose model in the backend and create a custom web interface in fewer than ten code lines. This web interface takes the SMILES and amino acid sequence as the input and returns prediction scores with less than 1-second latency. We provide examples in the Supplementary Material. 

*Financial Support*: none declared.M.Z. and K.H. are supported, in part, by NSF grant nos. IIS-2030459 and IIS-2033384, and by the Harvard Data Science Initiative. T.F. and J.S. was in part supported by the NSF SCH-2014438, IIS-1418511, CCF-1533768, IIS-1838042, the NIH award NIH R01 1R01NS107291-01 and R56HL138415.

*Conflict of Interest*: none declared.

## Supplementary Material

btaa1005_Supplementary_Data
