# Supplementary material for: DeepPurpose: a deep learning library for drug–target interaction prediction
Source: Bioinformatics. 2020 Dec 12;36(22-23):5545–7. doi: 10.1093/bioinformatics/btaa1005 (PMC8016467; doi:10.1093/bioinformatics/btaa1005)
Supplement: btaa1005_Supplementary_Data [file btaa1005_supplementary_data.zip › DeepPurpose_BIOINFO_SUPP (1).pdf]

Supplementary Information for: DeepPurpose: A Deep Learning Library for Drug-Target Interaction Prediction

Kexin Huang[1], Tianfan Fu[2], Lucas M. Glass[3], Marinka Zitnik[4], Cao Xiao[3], and Jimeng Sun[5,*]

[1]Health Data Science, Harvard T.H. Chan School of Public Health, Harvard University, Boston, MA 02115, USA
[2]College of Computing, Georgia Institute of Technology, Atlanta, GA 30332
[3]Analytic Center of Excellence, IQVIA, Cambridge, MA 02139, USA
[4]Department of Biomedical Informatics, Harvard University, Boston, MA 02115, USA
[5]Department of Computer Science, University of Illinois at Urbana-Champaign, Urbana, IL 61801, USA
[*]e-mail: jimeng@illinois.edu

ABSTRACT

Accurate prediction of drug-target interactions (DTI) enables drug discovery tasks, including virtual screening and drug repurposing, which can shorten the time to identify promising compound candidates and provide cures to patients. Recently, there is a growing number of research that developed deep learning (DL) models for DTI. Despite their superior performance, these research models are difficult to use in real drug discovery practice due to the complexity of deploying the research code as well as the restricted data formatting, model capacity, and evaluation setting. We present DeepPurpose, a comprehensive and easy-to-use deep learning library for DTI prediction. DeepPurpose enables training of customized DTI prediction models with 15 compound and protein encoders and over 50 neural architectures. To address the challenge of method performance evaluation and comparison, DeepPurpose provides a variety of data splits and automatic data loaders for popular benchmark datasets. We demonstrate state-of-the-art prediction performance of DeepPurpose on several benchmark datasets.

1 Further Details on DeepPurpose

Overview of DeepPurpose Framework. DeepPurpose uses an encoder-decoder framework for DTI prediction. The input of DeepPurpose is a compound SMILES string and protein amino acid sequence pair. The output of DeepPurpose is a score that measures the binding activity of the input compound protein pair. The power of deep learning models comes from its ability to create predictive feature vectors also called *embeddings*. In particular, DeepPurpose encodes both input compound and protein through various deep learning encoders to obtain their deep embeddings, then concatenates and feed them into a decoder, which is another deep neural network that aims to classify whether the input compound and target protein bind.

Encoders Implemented in DeepPurpose. DeepPurpose provides 8 compound encoders and 7 protein encoders with numerous variants, ranging from classic chemical informatics fingerprints to various deep neural networks. DeepPurpose feed two latent vectors generated from compound and protein encoders into the decoder to produce the final prediction score. With such a pipeline design, switching encoders is very simple in DeepPurpose. By configuring a different encoder name DeepPurpose will automatically switch to the required encoder model and connect them with the decoder for prediction.

Compound Encoders. The input compound is represented by SMILES strings corresponding to molecule graphs (Figure. 1).

1. **Morgan Fingerprint**[1] is a 1024-length bits vector that encodes circular radius-2 substructures. A multi-layer perceptron is then applied on the binary fingerprint vector.

2. **Pubchem**[2] is a 881-length bits vector, where each bit corrresponds to a hand-crafted important substructures. A multi-layer perceptron is then applied on top of the vector.

3. **Daylight** [1] is a 2048-length vector that encodes path-based substructures. A multi-layer perceptron is then applied on top of the vector.

[1]Daylight chemical information systems: https://www.daylight.com/

Ritonavir: CC(C)C1=NC(=CS1)CN(C)C(=O)NC(C(C)C)C(=O)NC(CC2=CC=CC=C2)CC(C(CC3=CC=CC=C3)NC(=O)OCC4=CN=CS4)O

SARS-CoV2-3CL Protease: SGFRKMAFPSGKVEGCMVQVTCGTTTLNGLWLDDVVYCPRHVICTSEDMLNPNYEDLLIRKSNHNFLVQA
GNVQLRVIGHSMQNCVLKLKVDTANPKTPKYKFVRIQPGQTFSVLACYNGSPSGVYQCAMRPNFTIKGSFLNGSCGSVGFNIDYDCVSFCYMHH
MELPTGVHAGTDLEGNFYGPFVDRQTAQAAGTDTTITVNVLAWLYAAVINGDRWFLNRFTTTLNDFNLVAMKYNYEPLTQDHVDILGPLSAQTG
IAVLDMCASLKELLQNGMNGRTILGSALLEDEFTPFDVVRQCSGVTFQ

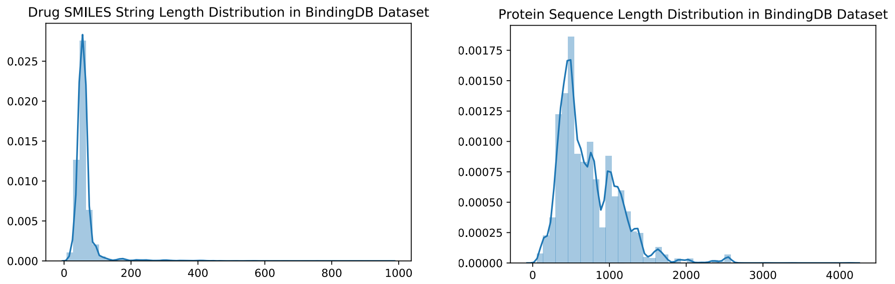

Figure 1. Examples of input representation. Compound is represented as SMILES string and protein is represented as amino acid sequence. The length distribution of the input are provided.

4. **RDKit-2D** [2] is a 200-length vector that describes global pharmacophore descriptor. It is normalized to make the range of the features in the same scale using cumulative density function fit given a sample of the molecules.
5. **CNN**[3] is a multi-layer 1D convolutional neural network. The SMILES characters are first encoded with an embedding layer and then fed into the CNN convolutions. A global max pooling layer is then attached and a latent vector describe the compound is generated.
6. **CNN+RNN**[4,5] attaches a bidirectional recurrent neural network (GRU or LSTM) on top of the 1D CNN output to leverage the more global temporal dimension of compound. The input is also the SMILES character embedding.
7. **Transformer**[6] uses a self-attention based transformer encoder that operates on the sub-structure partition fingerprint[7].
8. **MPNN**[8] is a message-passing graph neural network that operate on the compound molecular graph. It transmits latent information among the atoms and edges, where the input features incorporate atom/edge level chemical descriptors and the connection message. After obtaining embedding vector for each atom and edge, a readout function (mean/sum) is used to obtain a (molecular) graph-level embedding vector.

Protein Encoders. The input targets are proteins represented as sequences of 20 different kinds of amino acids (Figure. 1).

1. **AAC**[9] is a 8,420-length vector where each position correpsonds to an amino acid k-mers and k is up to 3.
2. **PseAAC**[10] includes the protein hydrophobicity and hydrophilicity patterns information in addition to the composition.
3. **Conjoint Triad**[11] uses the continuous three amino acids frequency distribution from a hand-crafted 7-letter alphabet.
4. **Quasi Sequence**[12] takes account for the sequence order effect using a set of sequence-order-coupling numbers.
5. **CNN**[3] is a multi-layer 1D convolutional neural network. The target amino acid is decomposed to each individual character and is encoded with an embedding layer and then fed into the CNN convolutions. It follows a global max pooling layer.
6. **CNN+RNN**[4,5] attaches a bidirectional recurrent neural network (GRU or LSTM) on top of the 1D CNN output to leverage the sequence order information.
7. **Transformer**[6] uses a self-attention based transformer encoder that operates on the sub-structure partition fingerprint[7] of proteins. Since transformer's computation time and memory is quadratic on the input size, it is computational infeasible to treat each amino acid symbol as a token. The partition fingerprint decomposes amino acid sequence into protein substructures of moderate sized such as motifs and then each of the partition is considered as a token and fed into the model.

[2]https: //github.com/bp-kelley/descriptastorus

Note on Feature-Architecture Combinations During implementation, we notice that all of the architectures require specific input features, which means other input features are incompatible with the architecture. DeepPurpose currently supports five architectures: MLP, CNN, CNN+RNN, Transformer, and MPNN, and it supports five types of features: fingerprints, SMILES string, ESPF fingerprint, and molecular graph. Notice that these architectures are not suitable for alternative features. MLP can take in fingerprints vectors, but one-hot matrices of SMILES or graphs are incompatible. CNN/CNN+RNN expect a matrix where each row is a one-hot vector for an entity in the SMILES/Amino Acids, but cannot take a single fingerprint vector or graphs. Transformer is quadratic to the input length, which makes the long fingerprints and SMILES/Amino acids computationally expensive. MPNN operates on graphs, thus, it is incompatible with fingerprints, SMILES, ESPF. In addition, the current included features encoders combinations are not randomly assembled but are previously included in the literature and have shown strong performance for molecular modeling with compounds and targets.

Programming Framework The functionality of DeepPurpose is modularized into six key steps where a single line of code can invoke each step: a) Load the dataset from a local file or load a DeepPurpose benchmark dataset. b) Specify the names of compound and protein encoders. c) Split the dataset into training, validation and testing sets using `data_process` function, which implements a variety of data-split strategies. d) Create a configuration file and specify model parameters. If needed, DeepPurpose can automatically search for optimal values of hyper-parameters. e) Initialize a model using the configuration file. Alternatively, the user can load a pre-trained model or a previously saved model. f) Finally, train the model using `train` function and monitor the progress of training and performance metrics.

Downstream Prediction, Objective Function, and Inference. After DeepPurpose obtains latent compound and protein embedding, both are fed into a multi-layer perceptron decoder. There are two classes of tasks/datasets in drug target interaction prediction. One's label is binding score such as Kd, IC50, and they are continuous values while the other one's label is binary, whether or not they can bind. DeepPurpose is able to automatically detect whether the task is regression for continuous label or classification for binary label by counting the number of unique labels in the data. For binding affinity score prediction, it uses mean squared error (MSE) loss (Eq. 1). For binary interaction prediction, it uses binary cross entropy (BCE) loss (Eq. 2).

$$\mathscr{L}_{MSE} = \frac{1}{n}\sum_{i=1}^{n}(\mathrm{y_i}-\hat{\mathrm{y}}_\mathrm{i})^2 \tag{1}$$

$$\mathscr{L}_{BCE} = \frac{1}{n}\sum_{i=1}^{n}\mathrm{y_i}\log(\hat{\mathrm{y}}_\mathrm{i})+(1-\mathrm{y_i})\log(1-\hat{\mathrm{y}}_\mathrm{i}), \tag{2}$$

where $\mathrm{y_i}$ is the true label and $\hat{\mathrm{y}}_\mathrm{i}$ is the predicted label for i-th compound-protein pair. For evaluation metrics, we use MSE, Concordance Index, and Pearson Correlation for continuous regression and Receiver Operating Characteristics-Area Under the Curve (ROC-AUC), Precision Recall-Area Under the Curve (PR-AUC) and F1 score at threshold 0.5 for binary classification. During inference, given new proteins or new compounds, the model prediction is used as the predicted binding score/interaction probability.

2 Demonstration of DeepPurpose for Drug-Target Prediction

Next, we show how to reproduce results shown in the main paper. In this example, we will use the CNN model to embed compounds and proteins from the DAVIS dataset[13]. We will train the CNN encoder model from scratch. Here are the necessary steps in DeepPurpose:

1. Start by using the `DeepPurpose.dataset` to load the DAVIS dataset:

```
>>> from DeepPurpose import DTI
>>> from DeepPurpose.utils import *
>>> from DeepPurpose.dataset import *
>>>
>>> X_drug, X_target, y = load_process_DAVIS(SAVE_PATH, binary=False)
```

2. Select the encoder models. Here, we use CNN to embed both compounds and proteins. Users can specify a variety of encoder models, which are implemented in DeepPurpose:

```
>>> drug_encoding, target_encoding = 'CNN', 'CNN'
```

3. Split data into training and test sets:

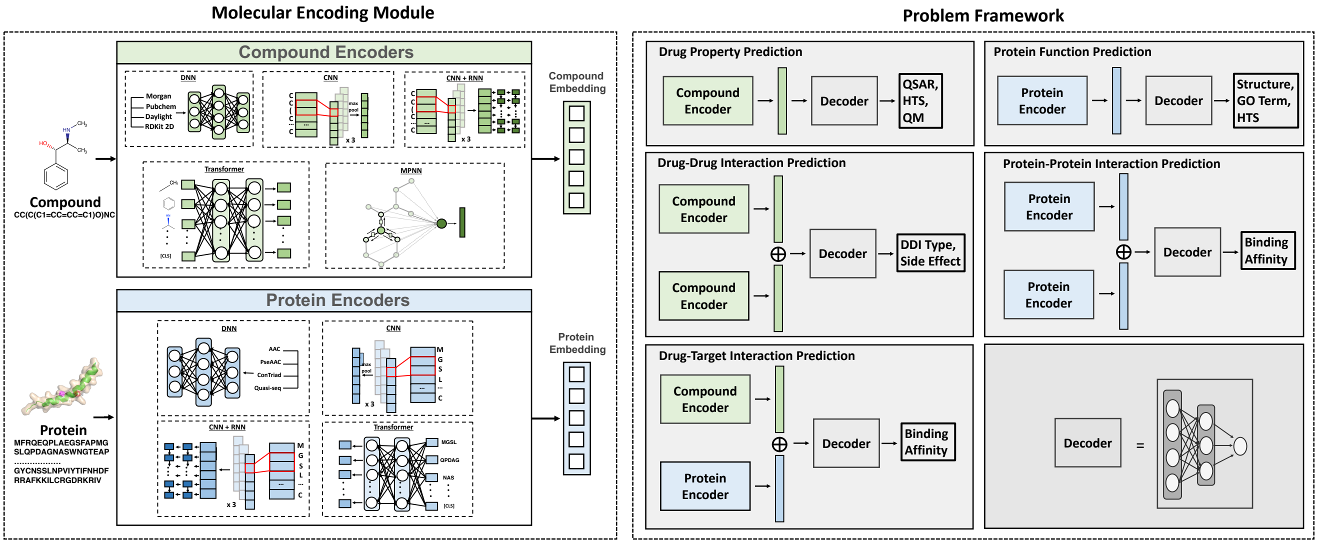

Figure 2. DeepPurpose supports DTI, DDI, PPI, Drug Property and Protein Function Prediction tasks.

```
>>> train, val, test = data_process(X_drug, X_target, y, drug_encoding, \
                                    target_encoding, split_method='random', \
                                    frac=[0.7,0.1,0.2], random_seed = 1)
```

4. Generate customized model configurations. Users can adjust model parameters and specify model hyperparameters. We use the DeepDTA's configuration[14]:

```
>>> config = generate_config(drug_encoding, target_encoding, \
                             cls_hidden_dims = [1024,1024,512], \
                             train_epoch = 100, LR = 0.001, batch_size = 256, \
                             cnn_drug_filters = [32,64,96], \
                             cnn_drug_kernels = [4,8,12], \
                             cnn_target_filters = [32,64,96], \
                             cnn_target_kernels = [4,8,12])
```

5. Initialize the model:

```
>>> model = models.model_initialize(**config)
```

6. Train the model. DeepPurpose prints the output of training process and saves testing metrics and figures in a directory with results:

```
>>> model.train(train, val, test)
```

Note that steps 2-6 are needed when we train deep models from scratch. Alternatively, we can use DeepPurpose's pre-trained models:

```
>>> models.model_pretrained(model = 'CNN_CNN_DAVIS')
```

DeepPurpose provides a variety of pre-trained models, which further increase the applicability of DeepPurpose package to downstream prediction tasks.

3 Illustration of DeepPurpose for Other Tasks Related to Compound and Protein

In addition to DTI prediction, DeepPurpose also supports user-friendly programming frameworks for other molecular modeling tasks, namely, drug/protein property prediction, drug-drug interaction prediction, protein-protein interaction prediction tasks. We provide a framework illustration in Figure. 2. The programming framework is similar to the DTI prediction illustrated above. We describe the sample programming codes below.

• **Drug Property Prediction:**

```
>>> from DeepPurpose import CompoundPred
>>> from DeepPurpose.utils import *
>>> from DeepPurpose.dataset import *
>>>
>>> X_drug, y = read_file_compound_property(PATH)

>>> drug_encoding = 'CNN'
>>> train, val, test = data_process(X_drug, y, drug_encoding, split_method='random', \
                                frac=[0.7,0.1,0.2], random_seed = 1)
>>> config = generate_config(drug_encoding)
>>> model = CompoundPred.model_initialize(**config)
>>> model.train(train, val, test)
```

• **Drug Drug Interaction Prediction:**

```
>>> from DeepPurpose import DDI
>>> from DeepPurpose.utils import *
>>> from DeepPurpose.dataset import *
>>>
>>> X_drug, X_drug_, y = read_file_training_dataset_drug_drug_pairs(PATH)

>>> drug_encoding = 'CNN'
>>> train, val, test = data_process(X_drug, X_drug_, y, \
                        drug_encoding, split_method='random', \
                         frac=[0.7,0.1,0.2], random_seed = 1)
>>> config = generate_config(drug_encoding)
>>> model = DDI.model_initialize(**config)
>>> model.train(train, val, test)
```

• **Protein Protein Interaction Prediction:**

```
>>> from DeepPurpose import PPI
>>> from DeepPurpose.utils import *
>>> from DeepPurpose.dataset import *
>>>
>>> X_target, X_target_, y = read_file_training_dataset_protein_protein_pairs(PATH)

>>> target_encoding = 'CNN'
>>> train, val, test = data_process(X_target, X_target_, y, \
                        target_encoding, split_method='random', \
                         frac=[0.7,0.1,0.2], random_seed = 1)
>>> config = generate_config(target_encoding)
>>> model = PPI.model_initialize(**config)
>>> model.train(train, val, test)
```

• **Protein Function Prediction:**

```
>>> from DeepPurpose import ProteinPred
>>> from DeepPurpose.utils import *
>>> from DeepPurpose.dataset import *
>>>
>>> X_target, y = read_file_training_dataset_protein_property(PATH)

>>> target_encoding = 'CNN'
>>> train, val, test = data_process(X_target, y, \
                        target_encoding, split_method='random', \
```

```
                           frac=[0.7,0.1,0.2], random_seed = 1)
>>> config = generate_config(target_encoding)
>>> model = ProteinPred.model_initialize(**config)
>>> model.train(train, val, test)
```

4 Illustration of using Gradio and DeepPurpose for Web UI

```
>>from DeepPurpose import utils
>>from DeepPurpose import DTI as models
>>import gradio

>>model = models.model_pretrained(model = 'MPNN_CNN_BindingDB')

>>def DTI_pred(drug, target):
>>    X_pred = utils.data_process(X_drug = [drug], X_target = [target], y = [0],
                                drug_encoding = 'MPNN', target_encoding = 'CNN',
                                split_method='no_split')
>>    y_pred = model.predict(X_pred)
>>    return str(y_pred[0])

>> gradio.Interface(DTI_pred,
                  [gradio.inputs.Textbox(lines = 5, label = "Drug SMILES"),
                   gradio.inputs.Textbox(lines = 5, label = "Target Amino Acid Sequence")],
                  gradio.outputs.Textbox(label = "Predicted Affinity")).launch(share=True)
```

References

1. Rogers, D. & Hahn, M. Extended-connectivity fingerprints. *J. chemical information modeling* **50**, 742–754 (2010).
2. Kim, S. *et al.* Pubchem 2019 update: improved access to chemical data. *Nucleic Acids Research* **47**, D1102–D1109 (2019).
3. Krizhevsky, A., Sutskever, I. & Hinton, G. E. Imagenet classification with deep convolutional neural networks. In *NeurIPS*, 1097–1105 (2012).
4. Cho, K. *et al.* Learning phrase representations using RNN encoder–decoder for statistical machine translation. In *EMNLP*, 1724–1734 (2014).
5. Hochreiter, S. & Schmidhuber, J. Long short-term memory. *Neural Computation* **9**, 1735–1780 (1997).
6. Vaswani, A. *et al.* Attention is all you need. In *NeurIPS*, 5998–6008 (2017).
7. Huang, K., Xiao, C., Hoang, T. N., Glass, L. M. & Sun, J. Caster: Predicting drug interactions with chemical substructure representation. *AAAI* (2020).
8. Gilmer, J., Schoenholz, S. S., Riley, P. F., Vinyals, O. & Dahl, G. E. Neural message passing for quantum chemistry. In *ICML*, 1263–1272 (2017).
9. Reczko, M. & Bohr, H. The DEF data base of sequence based protein fold class predictions. *Nucleic Acids Research* **22**, 3616 (1994).
10. Chou, K.-C. Using amphiphilic pseudo amino acid composition to predict enzyme subfamily classes. *Bioinformatics* **21**, 10–19 (2005).
11. Shen, J. *et al.* Predicting protein–protein interactions based only on sequences information. *Proc. Natl. Acad. Sci.* **104**, 4337–4341 (2007).
12. Chou, K.-C. Prediction of protein subcellular locations by incorporating quasi-sequence-order effect. *Biochemical and Biophysical Research Communications* **278**, 477–483 (2000).
13. Davis, M. I. *et al.* Comprehensive analysis of kinase inhibitor selectivity. *Nat. biotechnology* **29**, 1046 (2011).
14. Öztürk, H., Özgür, A. & Ozkirimli, E. Deepdta: deep drug–target binding affinity prediction. *Bioinformatics* **34**, i821–i829 (2018).
