# Supplementary material for: DeepPurpose: a deep learning library for drug–target interaction prediction
Source: Bioinformatics. 2020 Dec 12;36(22-23):5545–7. doi: 10.1093/bioinformatics/btaa1005 (PMC8016467; doi:10.1093/bioinformatics/btaa1005)
Supplement: btaa1005_Supplementary_Data [file btaa1005_supplementary_data.zip › DeepPurpose-Bioinfo.zip!/DeepPurpose_BIOINFO.pdf]

A. Molecular Encoding Module

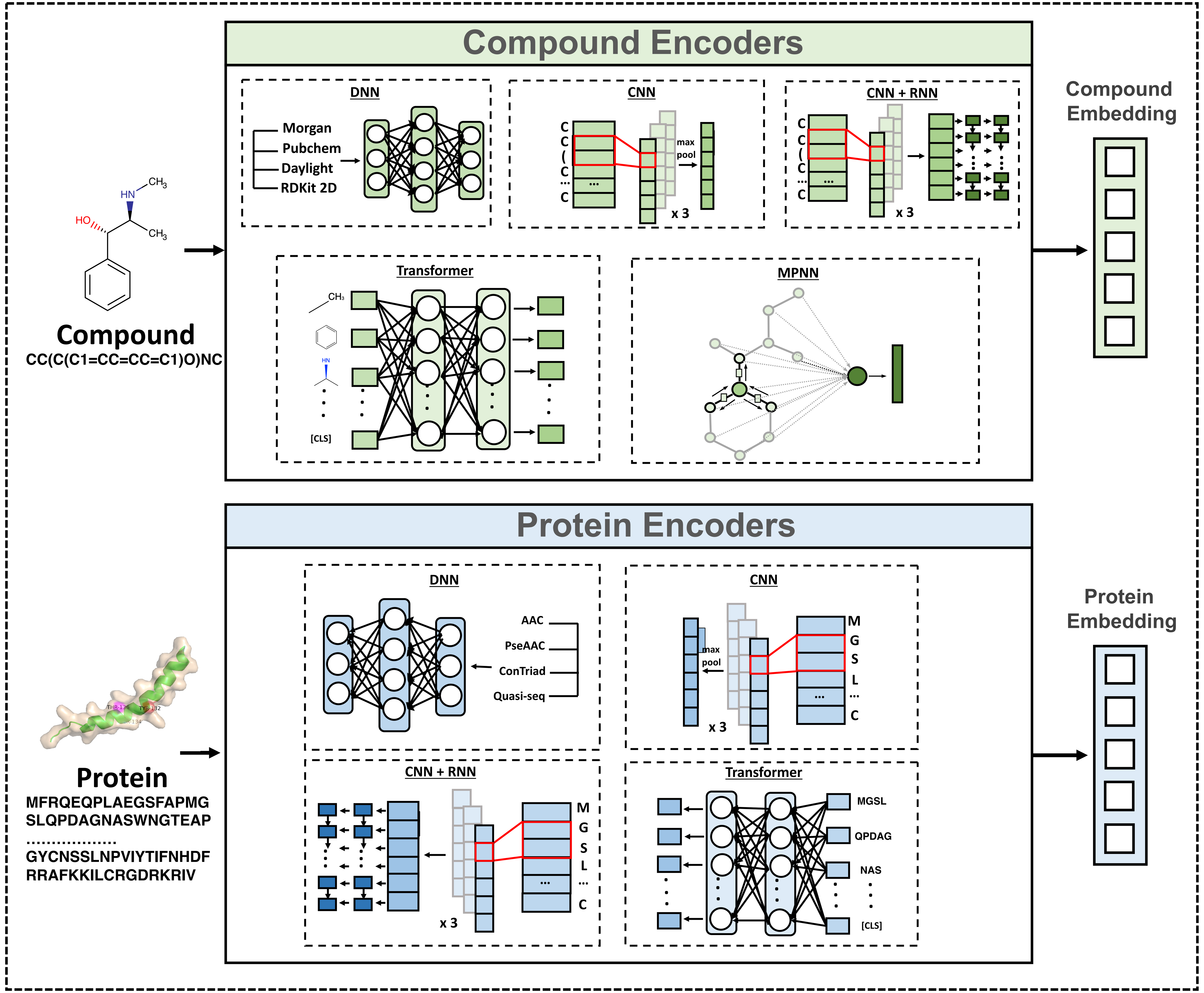

B. Drug-Target Interaction Prediction

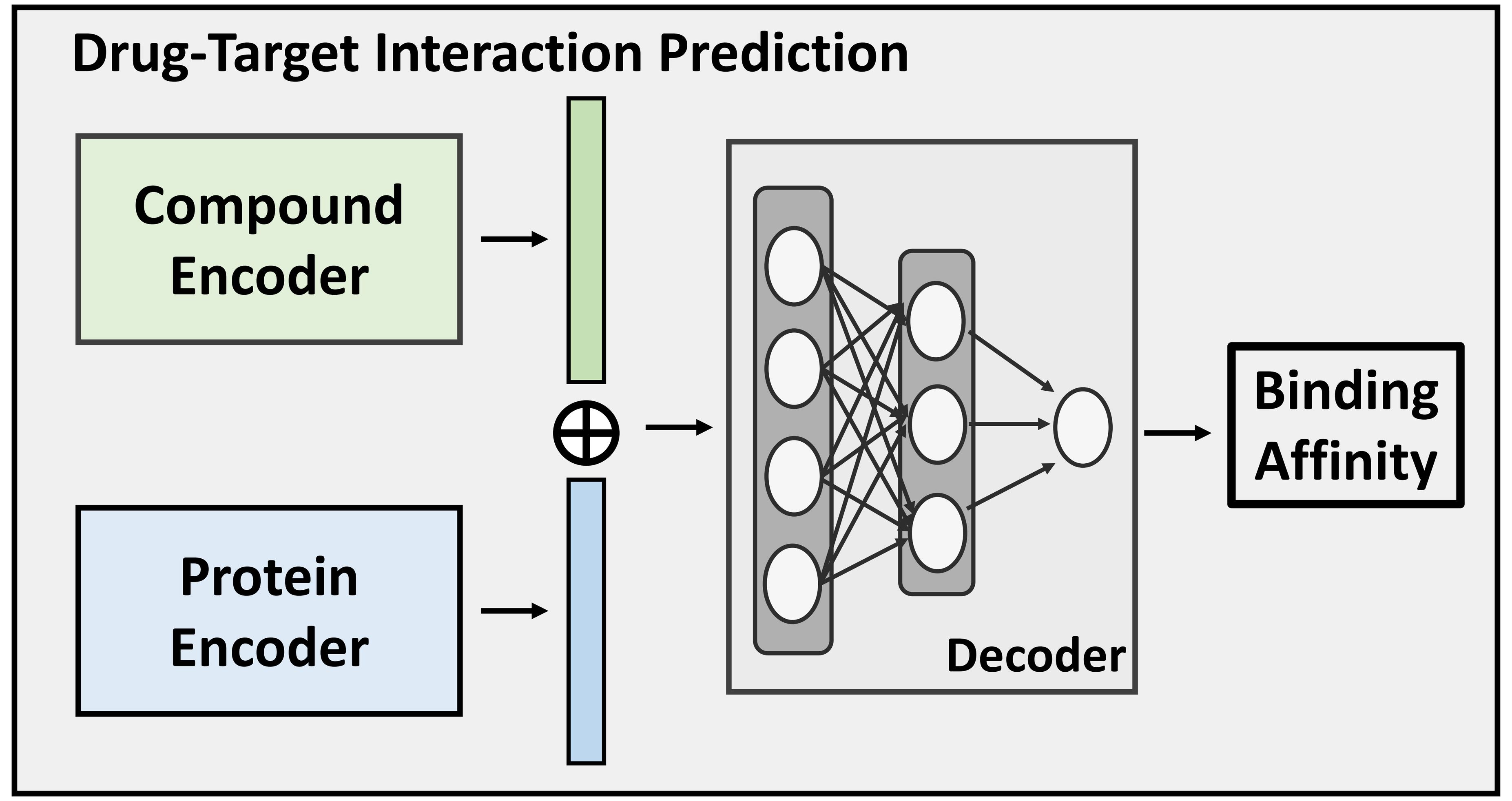

D. SOTA Prediction Performance

Dataset 1: DAVIS

	Model	MSE	Concordance Index
Baselines	KronRLS	0.329 (0.019)	0.847 (0.006)
	GraphDTA	0.263 (0.015)	0.864 (0.007)
	DeepDTA	0.262 (0.022)	0.870 (0.003)
DeepPurpose	CNN+CNN	0.254 (0.018)	0.879 (0.011)
	MPNN+CNN	0.271 (0.012)	0.858 (0.007)
	MPNN+AAC	0.242 (0.009)	0.881 (0.005)
	CNN+Trans	0.282 (0.009)	0.852 (0.006)
	Morgan+CNN	0.271 (0.012)	0.858 (0.007)
	Morgan+AAC	0.258 (0.012)	0.861 (0.008)
	Daylight+AAC	0.277 (0.014)	0.861 (0.008)

Dataset 2: KIBA

	Model	MSE	Concordance Index
Baselines	KronRLS	0.852 (0.014)	0.688 (0.003)
	GraphDTA	0.183 (0.003)	0.862 (0.005)
	DeepDTA	0.196 (0.008)	0.864 (0.002)
DeepPurpose	CNN+CNN	0.196 (0.005)	0.856 (0.004)
	MPNN+CNN	0.222 (0.006)	0.825 (0.003)
	MPNN+AAC	0.178 (0.002)	0.872 (0.001)
	CNN+Trans	0.240 (0.013)	0.818 (0.004)
	Morgan+CNN	0.229 (0.008)	0.825 (0.004)
	Morgan+AAC	0.233 (0.009)	0.823 (0.004)
	Daylight+AAC	0.252 (0.014)	0.808 (0.008)

C. DeepPurpose 10 Lines Framework

```
>>> from DeepPurpose import DTI
>>> from DeepPurpose.utils import *
>>> from DeepPurpose.dataset import *
>>>
>>> X_drug, X_target, y = load_process_DAVIS(SAVE_PATH, binary=False)
>>>
>>> drug_encoding, target_encoding = 'CNN', 'CNN'
>>> train, val, test = data_process(X_drug, X_target, y, drug_encoding, \
                                   target_encoding, split_method='random', \
                                   frac=[0.7,0.1,0.2], random_seed = 1)
>>>
>>> config = generate_config(drug_encoding, target_encoding, \
                             cls_hidden_dims = [1024,1024,512], \
                             train_epoch = 100, LR = 0.001, batch_size = 256, \
                             cnn_drug_filters = [32,64,96], \
                             cnn_drug_kernels = [4,8,12], \
                             cnn_target_filters = [32,64,96], \
                             cnn_target_kernels = [4,8,12])
>>>
>>> model = DTI.model_initialize(**config)
>>> model.train(train, val, test)
```

E. More Functionalities

- **Training Monitoring and Metrics Auto-generation**

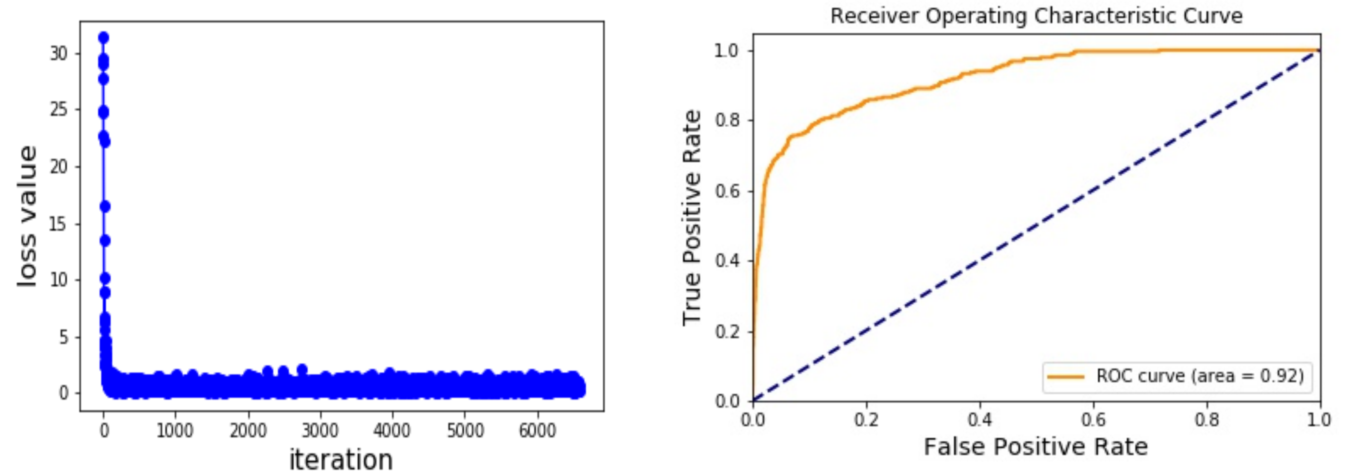

- **Repurposing and Screening Ranked List Generation**

Drug Repurposing Result for SARS-CoV2 3CL Protease

Rank	Drug Name	Target Name	Binding Score
1	Sofosbuvir	SARS-CoV2 3CL Protease	360.22
2	Daclatasvir	SARS-CoV2 3CL Protease	424.06
3	Vicriviroc	SARS-CoV2 3CL Protease	623.78
4	Efavirenz	SARS-CoV2 3CL Protease	768.33

- **Supporting Drug Property, Protein Function, Drug-Drug Interaction Prediction, and Protein-Protein Interaction Prediction, all less than 10 lines of codes.**
